# Total Facial Nerve Decompression for Severe Traumatic Facial Nerve Paralysis: A Review of 10 Cases

**DOI:** 10.1155/2012/607359

**Published:** 2011-11-20

**Authors:** Sertac Yetiser

**Affiliations:** Department of Otolaryngology, Head and Neck, Anadolu Medical Center, Kocaeli, 41400 Gebze, Turkey

## Abstract

Management of traumatic facial nerve disorders is challenging. Facial nerve decompression is indicated if 90–95% loss of function is seen at the very early period on ENoG or if there is axonal degeneration on EMG lately with no sign of recovery. Middle cranial or translabyrinthine approach is selected depending on hearing. The aim of this study is to present retrospective review of 10 patients with sudden onset complete facial paralysis after trauma who underwent total facial nerve decompression. Operation time after injury is ranging between 16 and105 days. Excitation threshold, supramaximal stimulation, and amplitude on the paralytic side were worse than at least %85 of the healthy side. Six of 11 patients had HBG-II, one patient had HBG-I, 3 patients had HBG-III, and one patient had HBG-IV recovery. Stretch, compression injuries with disruption of the endoneurial tubules undetectable at the time of surgery and lack of timely decompression may be associated with suboptimal results in our series.

## 1. Introduction

Indication and timing of the facial nerve decompression for facial paralysis and the anatomical extent of decompression has been a subject of controversy for years. Studies indicate that the number of surgical interventions has decreased over decades. In an analysis of large volume of published data between 1966 and 1999 regarding the management of facial nerve injury due to temporal bone trauma, Chang and Cass have reported that the patients with normal facial nerve function after injury regardless of progression, those with presentation of incomplete paralysis with no progression to complete paralysis, and those with less than 95% degeneration on ENoG at initial admission usually do not require surgical intervention. However, they have also reported that no data were available to provide information on exactly how much the return of function will be for the remaining patients who presumably have poorer prognosis [1]. Brodie and Thompson have reviewed 58 facial nerve injuries and reported that all patients with incomplete paralysis in the beginning recovered and 8 of 9 patients with delayed and 3 of 5 patients with sudden onset facial paralysis recovered after surgical decompression. But 2 of those (40%) patients with immediate-onset complete paralysis presented poor prognosis [2]. McKennan and Chole have compared recovery of patients with delayed and immediate-onset traumatic facial paralysis and have found that recovery is likely to occur in 94% of delayed-onset facial paralysis without surgical intervention [3]. Darrouzet et al. have reported that 49 of 50 medically treated patients based on clinical and electrophysiological assessment experienced normal or near-normal facial function recovery. They have reported that of the 65 surgically treated patients 52 had immediate paralysis and at 2 years after surgery, 93.8% had a grade-I–III recovery [4].

However, the issue of late exploratory surgery for those who do not experience adequate recovery of facial function also has many unclear points. Ulug and Ulubil have reviewed 10 patients with immediate-onset facial paralysis associated with temporal bone fracture who underwent surgical intervention ranging between 14 and 75 days after injury. They have reported HB-I recovery in 5 and HB-II recovery in 4 patients regardless of timing of surgery [5]. Quaranta et al. have studied 13 patients who underwent late decompression surgery for facial nerve paralysis due to temporal bone fracture and reported HB-I and II recovery in 78% of patients [6]. Sanus et al. have reviewed 25 patients with delayed traumatic facial nerve paralysis without temporal bone fracture who have worsening of facial function to complete paralysis. Of those, 13 patients underwent surgical decompression, whereas 12 patients were managed medically depending on clinical and electrophysiological findings, and complete or near complete recovery was found in 66.6% and 76.9% of patients in medically and surgically treated groups, respectively [7]. 

The aim of this study is to present retrospective review of 10 patients with complete facial paralysis after trauma who underwent total facial nerve decompression. 

## 2. Material and Methods

**Figure 1:**
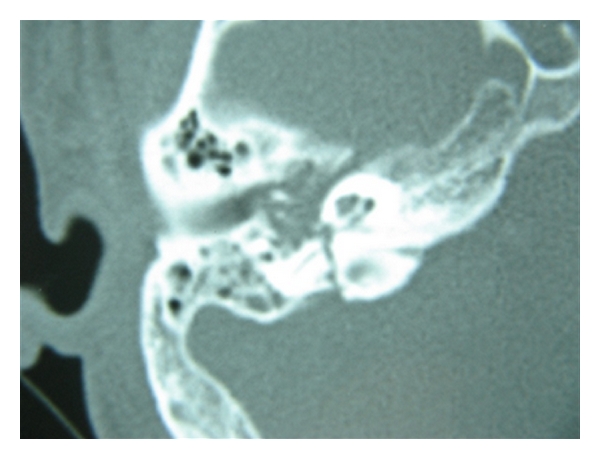
Transverse fracture of the temporal bone involving the midportion of the vertical segment is seen as an axial cut of the right temporal bone.

**Figure 2:**
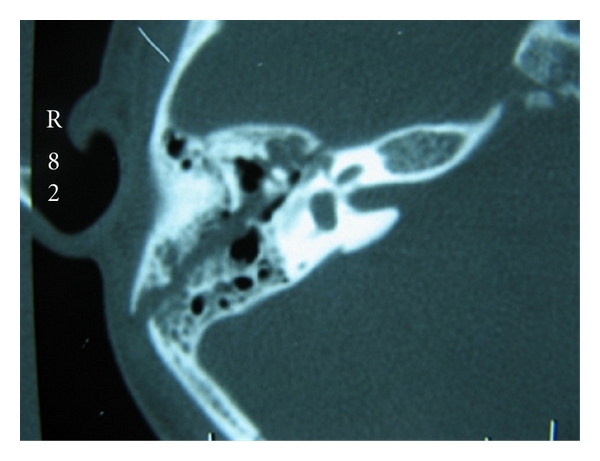
Longitudinal fracture of the temporal bone involving the perigeniculate region is seen as an axial cut of the right temporal bone.

Retrospective chart review of 10 patients who have undergone total facial nerve decompression due to severe traumatic facial paralysis between 2002 and 2010 were included. All patients had computerized tomography at the earliest. All patients had immediate-onset facial paralysis. House-Brackmann (HB) grading system was used to evaluate the function of the facial nerve [8]. Electromyography or electroneurography, if possible, was taken from the patients with facial paralysis. Excitation threshold, latency, and amplitude of orbicularis oculi muscle were used to compare normal and paralytic side during electroneurography. 9 patients are male, 1 patient is female with ages ranging from 20 to 53. 1 patient had bilateral and 9 patients had unilateral temporal bone fracture (5 right, 4 left). 2 patients had multiple and transverse, 1 patient had mixed (both parallel and perpendicular to the long axis of the petrous bone), and 7 patients had longitudinal fracture (Table 1, Figures 1 and 2).

Surgical technique: for patients with no hearing loss, transmastoid middle fossa combined approach was made with a postauricular skin incision at the mastoid apex going upward to the top of the auricle, 1.5–2 cm posterior and parallel to the postauricular sulcus. At 1 cm above the auricle, the incision was turned to the anterior for 3 cm, then following the temporalis hairy line, it was extended superiorly about 4 cm and then it was turned posteriorly for about 4 cm resembling “a reversed question mark.” Opposite to the skin incision (posteriorly based skin flap) an anteriorly based temporalis muscle flap incision was made for two-layer closure with inversely based opposing flaps at the end of surgery. After standard mastoidectomy and decompression of the facial nerve from digastric ridge to the second genu, a standard posterior tympanotomy was made, incudostapedial articulation was separated, and the incus was removed. Then the surgery was proceeded after drilling out the bone from tympanic side by decompressing the facial nerve anterior to the lateral semicircular canal from second genu to the geniculate ganglion beneath the malleus. At the level of head of malleus, 4 × 5 mm bone was removed from tegmen tympani to expose the dura to provide a landmark from middle fossa side. One cm above the mastoidectomy cavity, a 4 × 5 craniotomy parallel to the zygomatic route with the 2/3 rd of the base located anteriorly was made. Dura was retracted, bony opening landmark was located, and the facial nerve was decompressed from geniculate ganglion to the IAC. Dura over the IAC was cut to release some CSF. After total decompression, the sheath was cut along the nerve, steroid soaked gel foams was placed over the nerve, a piece of muscle was secured with fibrin glue over the IAC, and incus was articulated between the malleus and head of stapes in its original position and secured with glue. If the incus was dislocated or eroded lenticular process due to trauma, a partial prosthesis was placed between the ear drum and stapes. The wound closure was completed in a standard way.

For patients with total hearing loss, translabyrinthine approach was made with a skin incision from mastoid apex to the scalp going posterior for 5-6 cm; then the incision was turned to superior for 6 cm; then it was turned anterior again, toward the top of auricle parallel to the lower incision. Temporalis muscle incision followed the skin incision, and standard translabyrinthine approach was completed by decompressing the facial nerve totally from stylomastoid foramen to the IAC. After steroid moistened gel foam placement over the nerve, the cavity is filled with fat tissue and fixed with fibrin glue to prevent CSF leakage. 

## 3. Results

The type of trauma was as follows: falling from high in 2 patients, falling from bicycle in 1 patient, falling from behind a moving truck in 1 patient, traffic-car accident in 4 patients, strike of a flying heavy metal in dockyard in 1 patient, and explosion in 1 patient (Table 1). Facial nerve was totally exposed via middle fossa and transmastoid combined approach in 9 patients to preserve hearing and via translabyrinthine approach in 2 patients with total hearing loss due to transverse temporal bone fracture. One patient injured by explosion also had multiple fracture of the auditory canal and had canal wall down mastoidectomy during facial decompression. None of the patients had normal hearing before the surgery. Two patients had total hearing loss and 8 patients had conductive hearing loss. Out of 8 patients, 3 had profound (average air conduction between 62 and 55 dB), 2 had moderate (average air conduction between 55 and 30 dB), and 3 had mild (average air conduction between 30 and 20 dB) hearing loss.

Intraoperative pathology of the facial nerve was as follows: the integrity of the nerve was not interrupted as seen during surgery. Hematoma, multiple bone chips compression, granulation tissue, and edema were the main findings as summarized on Table 1. Extensive fibrosis around facial nerve was evident in patients with transverse fracture. Operation time after injury is ranging between 25 and 105 days (Table 2). Followup after surgery is ranging from 6 months to 3.5 years. Five patients had late EMG with axonal degeneration and 5 patients had electroneurography. None of the patients had voluntary motor unite action potential before the surgery. Excitation threshold, supramaximal stimulation, and amplitude on the paralytic side were worse than %85 of the healthy side in one patient and worse than 90% in the others. Two patients were judged as HBG-5 dysfunction before the surgery had total axonal degeneration at late EMG. During followup, all patients with MCF approach had audiogram. Electrophysiological evaluation was performed every 6 months. Three patients had partial prosthesis over the stapes, and 6 patients had incus relocation during surgery. One month after surgery average conductive hearing loss was 30 dB on the operated side, and 3 months after surgery patients had 22 dB average conductive hearing loss. No serious complication, including neurosensorial hearing loss and meningitis, was seen. Only one patient had normal facial function during followup. Six of 11 patients had HBG-II, and 3 patients had HBG-III (Table 2). 

## 4. Discussion

Electrical conduction may continue up to 72 hours to the muscles at the distal part of the injured nerve before a severe axonal block takes place. Facial nerve decompression and exploration are indicated if 90–95% loss of function is seen at the very early period on ENoG or if there is axonal degeneration on EMG lately with no sign of recovery. The latter is generally due to compression, edema, or intraneural hemorrhage without neural injury and usually does not necessitate surgery. However, early electrophysiological workout or even to evaluate the patient's voluntary facial movement is not possible often times in majority of patients with cranial trauma due to poor general condition at the emergency rooms and during intensive care period. Tests are neglected and treatment is delayed. The timing of surgical intervention is more than 30 days in 6 patients in this study. Priority of surgical intervention was modified due to multiple organ failures or the facial function was not properly evaluated because of loss of consciousness in these patients. 

Electroneurography performed in a few days after trauma is valuable to differentiate the severity of injury which will eventually result with HBG-1/II or HBG-VI. But it does not provide any information about the level of injury between HBG-II and VI. Therefore EMG is also valuable for the followup in the late period. However, predictive value of evoked EMG for traumatic facial paralysis has been found questionable in some studies. Sillman et al. have compared prognostic value of evoked EMG in 62 idiopathic and 29 traumatic facial paralysis. Of those 9 cases with idiopathic and 12 cases with traumatic facial paralysis underwent total nerve decompression as determined by maximal decline of compound muscle action potential (CAP). Among patients who did not undergo surgical decompression, incomplete clinical recovery was not associated with CAP decline of greater than 90% for traumatic paralysis [9]. Coker et al. have proposed that excitation threshold below 3.5 mA on the paralytic side is a worse prognostic sign [10].

The incidence of temporal bone trauma and associated facial nerve injury has increased in recent decades together with the increasing traffic and population [11]. Management of traumatic facial nerve disorders is challenging. The type of injury, sudden or delayed-onset, complete or partial paralysis, localization of the injury, and severity of conduction block based on the electrophysiological tests are the main determinants of the prognosis. Cranial injury may or may not be with temporal bone fracture and it is difficult to tell that in which type of fracture, the axis has greatest risk to intervene with the course of the nerve. Coker et al. have reported that 14 of 18 patients with temporal bone fracture who needed to have facial nerve exploration had longitudinal fracture [10]. Ulug and Ulubil have reported that 7 of 11 fractures in their surgical treatment series were of longitudinal type [5]. Majority of the patients in our series had longitudinal fracture which was associated with the involvement of the fallopian canal in the perigeniculate region. Labyrinthine segment is the most delicate and narrow part of the facial nerve. Degenerative and fibrotic changes after severe injury affect this region more than any part of the facial nerve. Felix et al. have examined facial nerve segments removed from 12 patients with persisting facial paralysis following temporal bone fracture and found that traumatic injury involving the geniculate ganglion presented profound retrograde degeneration through the labyrinthine and distal meatal segments of the facial nerve even though the fracture line was involving the horizontal segment only [12]. Hematoma, multiple bone chips compression, and edema were the main findings in our patients as seen in Table 1. Extensive fibrosis around facial nerve was evident in patients with transverse fracture. However, the integrity of the nerve was not interrupted as seen during surgery except in one patient with gunshot wounding who has grafting with greater auricular nerve. 

Middle cranial or translabyrinthine approach is planned for total nerve exploration depending on hearing. Horizontal segment and geniculate ganglion can be exposed via transmastoid transattical approach [13–15]. However, superior canal and its ampulla limit the exposure to the labyrinthine segment from transmastoid approach. Nyberg and Fisch, and later on Graham and Kemink, described transmastoid and middle fossa combined total facial nerve exploration in patients with recurrent facial paralysis [16]. Total facial nerve decompression instead of limited segmental access to the blocked motor fibers is preferred in our series. This approach provides inspection of the facial nerve in every segment from brainstem to the parotid [17, 18]. We always attempt to do posterior tympanotomy to inspect the middle ear and prefer to remove incus before decompressing the horizontal segment to avoid the vibratory hazardous effect of the drill to the ossicular chain. The incus is later secured in its original position with some bone cement and fibrin glue. Mild conductive hearing loss was restored within 3 months after surgery and none of the patients with middle fossa approach had severe conductive or neurosensorial hearing loss.

The rate of recovery within HBG I-II after total facial nerve exploration in our short series is 70% (10/7). Stretch, compression injuries with disruption of the endoneurial tubules undetectable at the time of surgery may be associated with suboptimal results in our series. One other possible explanation would be the lack of timely decompression of the facial canal in some of them. 

## Figures and Tables

**Table 1:** Intraoperative findings of facial nerve (TA: traffic accident, LF: longitudinal fracture, TL: translabyrinthine, MCF: middle cranial fossa, FD: falling down, HMT: heavy metal hit, Exp: explosion, FB: falling from bicycle, FT: falling from truck, RE: right ear, and LE: left ear).

No	Cause	Site	Type of fracture	Operation	Facial nerve
(1)	TA	RE	LF	TL	Labyrinth filled with fibrous tissue
(2)	TA	RE	LF	MCF	Compression of genu with bony fragment
(3)	FD	LE	LF	MCF	Hyperemia and edema of geniculate ggl.
(4)	FD	LE	LF	MCF	Edema, granulation tissue
(5)	HMH	RE	LF	MCF	Hyperemia of geniculate ganglion
(6)	Exp	LE	TF	TL	Extensive edema, granulation tissue of the vertical segment
(7)	FB	RE	LF	MCF	Granulation tissue, fibrosis
(8)	FT	Bilat	Bilat LF	MCF (RE)	Fractured fragments
(9)	TA	RE	TF	MCF	Granulation tissue throughout the vertical segment of the nerve
(10)	TA	LE	LF	MCF	Granulation tissue

**Table 2:** Electrophysiology, surgical timing and the results of facial decompression (Mo: month, OOc: orbicularis oculi, mV: microvolt, mA: milliampere, SMS: supramaximal stimulation, amp: amplitude, ET: excitation threshold, RE: right ear, LE: left ear, and MUP: motor unite potential).

No	EMG/ENoG	Loss %	Timing	Preop grade	Postop grade
(1)	Total axonal degeneration	—	1 mo	6	2
(2)	Total axonal degeneration	—	1.5 mo	5	2
(3)	Total axonal degeneration	—	2 mo	5	1
(4)	OOc; SMS RE; 25, LE; 100 mA ET RE; 17, LE; 2.1 mA No voluntary MUP	89%	1.5 mo	6	2
(5)	OOc; amp RE; 0.2, LE; 2 mV No voluntary MUP	90%	2 mo	6	3
(6)	OOc; amp RE; 2.3, LE; 0.2 mV No voluntary MUP	91%	25 days	6	2
(7)	OOc; amp RE; 0.3, LE; 2.8 mV ET RE; 44, LE; 6.4 mA No voluntary MUP	85%	1 mo	6	2
(8)	OOc; amp LE; 2.5, RE; 0.2 mV No voluntary MUP	92%	1 mo	6	2
(9)	Total axonal degeneration	—	3.5 mo	6	3
(10)	Total axonal degeneration	—	2 mo	6	3
